# Progress in Cancer

**Published:** 1904-04-16

**Authors:** 


### Progress in Cancer.

*(Concluded from page 29.)*

**The Latest Views on Cancer.**[12]—Under this heading appears a very brief review of a number of papers devoted to this subject occurring in the *British Medical Journal* for December 12th. The first is Mr. Morris's Bradshaw lecture on "Cancer and its Origin." The various theories as to the origin of both carcinoma and sarcoma are reviewed. The theory the author himself believes destined to be accepted as the true explanation of the genesis of malignant growths is that put forward by Durante about the year 1874, and a year or two later by Cohnheim. He deems this theory more consistent than any other with all that we know about malignant diseases and which fully explains the origin of the very many non-malignant tumours. Cohnheim's view was that the origin of carcinoma was due to the proliferation, not of mature but of embryonic epithelial cells which during fœtal life had been cut off from their proper connections and remained in an undeveloped state as "embryonic inclusions." The deficiency of this theory, according to Mr. Morris, is that it does not explain the formation of malignant growths in scar tissue, in immature callus, in unabsorbed inflammatory products and in parts after injuries and operations of various kinds. To meet this deficiency Senn added the following corollary: "The tumour matrix may be composed of embryonic cells, the offspring of mature cells which have failed to undergo transformation into higher tissue, and which may remain in a latent, immature state for an indefinite period of time, to become, under hereditary or acquired exciting causes, the essential starting point of a tumour." Mr. Morris examines separately the evidence in favour of the separate parts of the "tumour germ" theory, namely: 1. The existence of congenital matrices of embryonic cells. 2. The existence of matrices of post-natal formation. 3. The continuance of these matrices for a time in a dormant state. 4. The causes which arouse the matrices into activity. He makes out a strong case for the tumour-germ theory. He also points out that the researches for some years past have been directed too exclusively to microbes and too little to cancer. He is of the opinion that the microbic theory of the origin of cancer is decidedly losing ground. Plimmer contributes a paper on the parasitic origin of cancer. Among other strong evidence in support of this theory he mentions Behla's view that cancer is frequently conveyed by uncooked infected vegetables. Dr. Lyon's figures with regard to cancer in Buffalo are also quoted as confirmatory of this idea. Statistics roughly showed that for the same population the cases of cancer in the German wards of Buffalo were double the number of those in the native, due, in Dr. Lyon's opinion, to the fact that the Germans were in the habit of eating uncooked vegetables. Many other points tending to substantiate the parasitic theory are advanced by the writer. Mr. Lenthal Cheatle contributes an article with many illustrations, in which he discusses the behaviour of cancer within nerve and trophic areas and is almost confirmed in the truth of a suggestion he put forward some time ago, viz., that a possible direct or indirect nervous influence is the chief agent in directing the areas occupied by cancer, and is responsible for its spread. His reason for this belief, amongst others, is that it is impossible to omit the subject of irritation from amongst the etiological factors of cancer. Dr. Arthur Newsholme, in his article, endeavours to trace the possible association of the consumption of alcohol with excessive mortality from cancer. Many figures are quoted, and the writer sums up by saying "that evidently, however, if alcohol exerts an irritating effect, this is only one of a number of conditions favouring cancer, and alcohol cannot be elevated to the position of a primary cause.

**Late Marriages a Possible Cause of the Increase**

**of Cancer.**[13]—This question is raised in an editorial in the *Polyclinic*, and it is suggested that the nearer to the cancer period in the parent a child is begot the greater is the risk that he may inherit a tissue proclivity to the cancerous process, and that further, if we suppose two communities, in one of which marriage shall be prohibited to men until the age of fifty, whilst in the other it shall be encouraged at eighteen, that in the former there would ensue a definite tendency to the increase of cancer. In some support of this theory it is pointed out that cancer is far less common in the lower animals than in man, and they, as a rule, wed early. In man it is less common in lowly-civilised communities than it is amongst others. Its recent increase has been noted exclusively amongst the more highly civilised nations and it is in them that for some long time the age at which marriage occurs has been steadily rising. It is even possible that religious creed may have something to do with the results. Roman Catholicism encourages early marriage, and there are some facts which might seem to show that there is less cancer amongst its adherents than among Protestants. The chief interest of the theory seems to lie in the fact that it is one capable of definite proof or disproof.

**Radium in Cancer.**—Macintyre[14] discusses the properties of radium and the results of treatment with it, and concludes that the indications point to its being useful in the same class of affections as are at present being treated with *x*-rays, Finsen light, or high-frequency currents. Davidson[15] reports a number of cases treated with radium bromide, and finds that whereas the emanations produce profound changes in rodent cancer, it is with the present method of application of no use whatever in carcinoma.

**The Treatment of Cancer by Injections of Sodium Cinnamate.**[16]—Lovel Drage reports several cases treated in this way. He sums up as follows: The condition which the treatment is designed to produce is first, a leucocytosis; and secondly a fibrosis in the tissue affected by cancer. In a case of pulmonary tuberculosis there is no difficulty in producing these conditions; in that of cancer much greater difficulty is experienced in advanced cases than in the case of tuberculosis. However, it is claimed that the treatment is soundly based, and that, although it is quite likely that the best possible means of initiating the changes desired has not yet been found, it is only a matter of time for improvement in the details of treatment to be effected.

**A Case of Sarcomatosis Cutis.**—Wilson and Ralteyer[17] relate this case. It was a young woman, aged 26, who had a pigmented mole on the forehead, which grew rapidly after an injury, was removed by operation, and a normal scar formed. About the time of operation, and shortly afterwards, disseminated nodules formed in the skin, and subcutaneous tissues with rapid enlargement of the liver and spleen. Wasting, and a fatal termination followed. The tumour removed from the forehead was dark purple, soft and very vascular. The secondary nodules were small, the largest a centimetre in diameter. They were tender, firm, and presented no evidences of inflammation. They were most numerous on the chest and abdomen, where there were twenty-five. Some of the tumours had a bluish colouration, though the skin over most of them was not discoloured. The liver was enlarged irregularly, and reached four fingers' breadth below the ribs. Wasting occurred very rapidly, and vomiting was a prominent symptom. Microscopic examination of one of the tumours removed during life showed it to be a spindle-celled melanotic sarcoma. Unna's view that malignant melanotic growths arising from moles or nævi are of epithelial origin, and not sarcomatous, is discussed, and the authors think it perhaps cannot yet be definitely settled, but state that their case was undoubtedly sarcomatous. A brief review of fifty cases of multiple sarcomata from recent literature follows. These are divided into three groups: 1, multiple melanotic sarcomata; 2, multiple non-pigmented sarcomata; and 3, multiple pigmented hæmorrhagic sarcomata. The treatment of these cases is most unsatisfactory, and although many instances may be found where excision is not followed by recurrence, as a rule it fails in influencing the rapid metastases which follow. Whitehead advances the following conclusions: 1, a mole on the foot of a middle-aged person should be excised; 2, an ulcer beginning in a mole on the foot demands immediate and radical surgery; 3, the prognosis of ulcerated moles should be guarded even where the growth seems non-malignant. In conclusion, attention is drawn to the great danger that a mole may take on active growth, and to the importance of early operation in those tumours which spring from pigmented moles.

[12] Med. Rec., Jan. 16, 1904. [13] Scot. Med. and Surg. Jour., Oct. 1903. [14] Brit. Med. Jour., Dec. 12, 1903. [15] Ibid., Jan. 23, 1904. [16] Ibid., Dec. 12, 1903. [17] Amer. Jour. Med. Sc., Nov. 1903.

